# Supplementary figures and images for: Common Genetic Aberrations Associated with Metabolic Interferences in Human Type-2 Diabetes and Acute Myeloid Leukemia: A Bioinformatics Approach
Source: Int J Mol Sci. 2021 Aug 28;22(17):9322. doi: 10.3390/ijms22179322 (PMC8431701; doi:10.3390/ijms22179322)

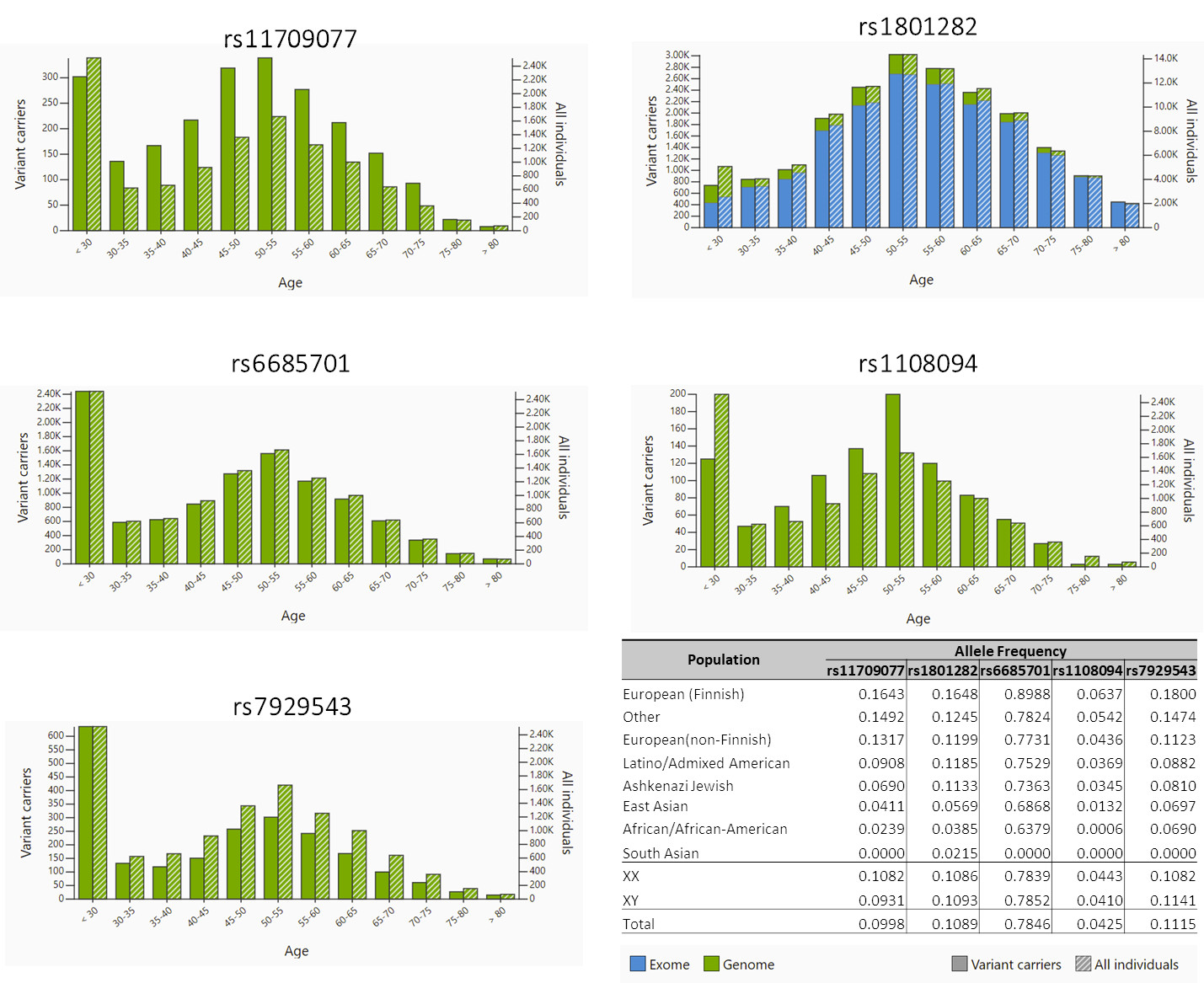

Supplement: Supplementary file 1 [file ijms-22-09322-s001.zip › ijms-1302087-supplemenatary/ijms-1302087-supp-fig.jpg]
